# To Investigate the Effect of Colchicine in Prevention of Adhesions Caused by Serosal Damage in Rats

**DOI:** 10.1155/2015/315325

**Published:** 2015-09-28

**Authors:** İhsan Yıldız, Yavuz Savas Koca

**Affiliations:** Department of General Surgery, School of Medicine, Suleyman Demirel University, Isparta, Turkey

## Abstract

*Introduction and Aim*. Adhesion formation is a process which starts with an inflammation caused by a number of factors and eventually results in fibrosis. Colchicine prevents adhesion formation which is antifibrous process. The effectivity of colchicine in the prevention of adhesions was investigated. *Materials and Methods*. A total of 36 rats were equally divided into three groups: (I) control group 1 (*n* = 12), (II) abrasion group 2 (*n* = 12), and (III) abrasion + colchicine group 3 (*n* = 12). Group 1 underwent laparotomy and was orally given physiological serum 2 cc/day for 10 days. In Group 2, injury was created in the cecum serosa following laparotomy and they were orally given physiological serum 2 cc/day for 10 days. In Group 3, injury was created in the cecum serosa following laparotomy and the rats were orally given colchicine 50 mcg kg/day mixed with physiological serum 2 cc/day for 10 days. Laparotomy was performed and adhesions were examined both macroscopically and microscopically. Both macroscopic and microscopic examinations were performed using Zühlke's score. *Results*. A significant difference was observed among the adhesion scores of the groups both macroscopically and microscopically. Macroscopic score was lower in group 3 than group 2. Microscopic score was lower in group 3 than group 2. *Conclusion*. Oral administration of colchicine is effective in the prevention of adhesions.

## 1. Introduction

Intraperitoneal adhesions remain a challenging surgical problem. These adhesions often cause a number of clinical problems including intestinal obstruction, chronic abdominal pain, and infertility. During abdominal surgery, serosal adhesions may lead to organ injuries and thus may lead to higher morbidity and mortality. For a long period of time, numerous studies have been conducted to investigate the pathophysiology of adhesion formation and to prevent adhesion formation in light of the results obtained [1–9]. Adhesion formation starts with the accumulation of fibrin matrix, which becomes organized and eventually results in fibrous adhesions within 5–7 days. Postsurgical adhesions occur among the traumatic serosal surfaces induced by the trauma during surgery. Wound healing process starts when the inflammation following tissue injury occurs [3]. During the wound healing process, a gel fibrin structure oozes out and if this structure is not destroyed by the fibrinolytic activity, a fibrin clot is formed, leading to permanent fibrous bands [4, 5].

Colchicine is a natural drug which provides various effects including antifibrotic activity, anti-inflammatory effect, membrane stabilization, and the inhibition of lipid peroxidation. Due to these effects, colchicine is considered to be effective in the diseases closely associated with inflammatory events and cell division [6]. Colchicine inhibits the macrophage release of fibronectin and the release of growth factor from the macrophages and also suppresses the cellular replication by connecting to the tubulin and the release of cytokines from the polymorphonuclear leukocytes. Pharmacodynamic studies have demonstrated that the biological effect of colchicine is associated with the plasma concentration. Colchicine exerts antimitotic effect in a dose-dependent manner. This effect takes 30–120 min to occur. The oral bioavailability of colchicine is 50%. The elimination half-life of colchicine ranges between 20 and 40 h. Most common side effects of colchicine include diarrhea, nausea, and vomiting [4, 5].

Colchicine prevents adhesion formation during the healing process of serosal injury by altering the neutrophil migration and the distribution of adhesion molecules on neutrophils and endothelial cells [5]. The aim of this experimental study was to investigate the effectivity of colchicine in the prevention of adhesions induced by serosal injury.

## 2. Materials and Methods

The study included 36 young adult Wistar Albino rats weighing 210–280 g. The rats were divided into three groups as follows: (I) control group (*n* = 12), (II) abrasion group (*n* = 12), and (III) abrasion + colchicine group (*n* = 12). Intramuscular anesthesia was performed using 80 mg/kg ketamine hydrochloride (Ketalar, Parke-Davis, Morris Plains) and 10 mg/kg xylazine hydrochloride (Rompun, Bayer). All the surgical procedures were performed in sterile conditions. In all the rats, the abdominal skin was shaved and cleaned with povidone-iodide solution. The surgical site was covered with green sterile drape with a hole. Laparotomy was performed through a 3-cm midline incision. The cecum and the terminal ileum were localized and wet gauze was placed on them. Abrasion was performed on the antimesenteric surface of the cecum using dry gauze. The procedure was continued until petechial bleeding foci were seen on the surfaces (cecal abrasion model).

### 2.1. Groups

The control group underwent laparotomy only and was orally given physiological serum 2 cc/day for 10 days. In the abrasion group, injury was created in the cecum serosa following laparotomy. This group was also orally given physiological serum 2 cc/day for 10 days. In the abrasion + colchicine group, injury was created in the cecum serosa following laparotomy and the rats were orally given colchicine 50 mcg/kg/day with physiological serum 2 cc/day for 10 days (using gavage).

**Figure 1:**
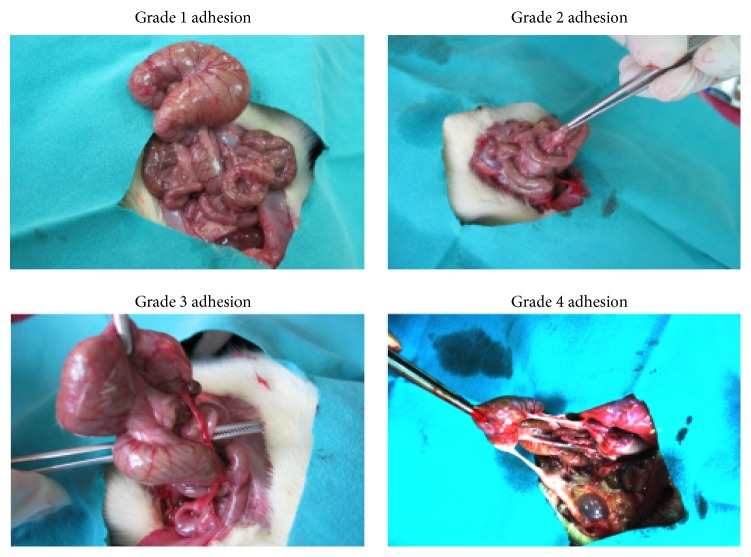
Macroscopic scoring of adhesions.

Laparotomy was performed on postoperative day 10 in order to monitor and score the adhesions. The adhesions were examined both macroscopically (see the Zühlke Macroscopic Classification, Figure 1) and microscopically (see the Zühlke Microscopic Classification) using Zühlke's grading system.

*The Zühlke Macroscopic Classification.* The classification is as follows:

 Grade 1: filmy and easy to separate by blunt dissection.
 Grade 2: blunt dissection possible, partly sharp dissection necessary, and beginning vascularization.
 Grade 3: lysis possible by sharp dissection only, clear vascularization.
 Grade 4: lysis possible by sharp dissection only, organs strongly attached with severe adhesions, and damage of organs hardly preventable.

*The Zühlke Microscopic Classification.* The classification is as follows:

 Grade 1: loose connective tissue, cell-rich, old and new fibrine, fine reticular fibers.
 Grade 2: connective tissue with cells capillaries, few collagen fibers.
 Grade 3: connective tissue more firm, fewer cells, more vessels, and few elastic and smooth-muscle fibers.
 Grade 4: old firm granulation tissue, cell-poor, serosal layers hardly distinguishable.

The crude drugs were examined under a light microscope and then photographed. The results were evaluated using the microscopic classification system developed by Zühlke (Figure 1).

Statistical analyses were performed using Mann-Whitney *U* Test. A *p* value of <0.05 was considered significant.

## 3. Results

No subjects were lost and no healing problems such as infection and delayed wound closure occurred in any group. No side effects of colchicine such as diarrhea, hair loss, and bone marrow depression were detected. No serious feeding disorder, inappetence, or weight loss was observed in the postoperative period.

The macroscopic adhesion scores were significantly higher in the abrasion and abrasion + colchicine groups compared to the control group (*p* < 0.01 and *p* < 0.05, resp.) (Table 1). Similarly, the microscopic adhesion scores were significantly higher in the abrasion and abrasion + colchicine groups compared to the control group (*p* < 0.01 and *p* < 0.05, resp.) (Table 2).

The macroscopic adhesion scores were significantly higher in the abrasion group compared to the abrasion + colchicine group (*p* < 0.01) (Table 1). Likewise, the microscopic adhesion scores were significantly higher in the abrasion group compared to the abrasion + colchicine group (*p* < 0.01) (Table 2).

## 4. Discussion

Adhesions occur in 90% of the patients undergoing intra-abdominal surgery and they lead to intestinal obstruction in 3% of these patients. These adhesions often cause numerous clinical problems including mechanical intestinal obstruction, infertility, and chronic abdominal pain [2–6]. However, it is difficult to predict when adhesions may cause intestinal obstruction. The experimental model used in this study was a cecal abrasion model which mimics the trauma caused by laparotomy. However, the absence of postoperative symptoms such as inappetence, nausea, and vomiting in our rats was attributed to the absence of adhesion-induced obstruction.

Numerous studies have been conducted to prevent intraperitoneal adhesions [2–14]. These studies have utilized various substances including the mechanic effects of solid-liquid and organic barriers, fibrinolytic agents, anticoagulants, and antibiotics [2–14]. In order to prevent adhesions, surgeons have investigated the methods of migrating the fibrin glue from the peritoneum and have tried a number of techniques including peritoneal washing, dilution of the fibrin glue, melting of the fibrin gel with hyaluronidase gel or fibrinolysin, and enzymatic digestion of the fibrin glue; however, limited success has been obtained [4].

Tarhan et al. reported that a fibrinolytic activator was detected on the mesothelial surfaces and the activator exhibited a significant decrease following the creation of serosal trauma with phenol and formaldehyde [10]. As a result of the decrease in the serosal fibrinolytic activation system, intra-abdominal adhesions develop approximately on day 10 after the decrease [3]. Since the fibrin is not destroyed due to the reduction of tissue plasminogen activator (t-PA) release, fibrinous adhesions occur. These adhesions then become organized and turn into fibrous adhesions [4, 5]. Depending on this process, we performed laparotomy on day 10 in order to monitor adhesion formation.

Dargenio et al. compared the effect of colchicine and dexamethasone in the prevention of adhesions and reported that colchicine yielded better outcomes [12]. The study administered the colchicine intramuscularly or intraperitoneally, whereas we administered it orally. Ince et al. conducted a dose-dependent experiment on rabbits and used various doses of colchicine in the prevention of adhesions caused by intraperitoneal infection [4]. The study reported that the response obtained with a dose of 1 mg/kg was sufficient for the prevention of the adhesions. Rojkind and Kershenobich, demonstrating the colchicine as a collagen synthesis inhibitor, used colchicine in the prevention of fibrosis in liver cirrhosis and obtained successful outcomes in 5- and 10-year follow-ups [13]. Similarly, Ben-Chetrit et al. investigated the anti-inflammatory effects of colchicine in rheumatological patients in 2006 and obtained successful results [5]. On the other hand, Granat et al. reported that the intraserosal administration of colchicine 50 mcg/day provided better results when mixed with dexamethasone [7]. In our study, colchicine was administered orally at a dose of 50 mcg/kg/day.

In our study, the highest adhesion score was in the abrasion group, followed by the colchicine and control groups, respectively. The presence of the lowest score in the control group was attributed to the absence of cecal abrasion in this group. In the abrasion + colchicine group, colchicine was highly effective.

The results suggested that colchicine is an effective agent in the prevention of adhesions. Colchicine is considered to inhibit collagen synthesis by restraining the mitotic activity and fibroblastic response and thus prevent the formation of fibrotic adhesions which lead to obstruction. As mentioned above, since colchicine inhibits the mitotic activity, it also reduces the activity of the macrophages as well as macrophage migration and thus prevents inflammation. In this study, the macroscopic parameters were analyzed but no evaluation was performed for the phagocytic activities of the macrophages. Therefore, further studies are needed to perform an extensive analysis on the effects of colchicine in the prevention of adhesions and an evaluation on the inflammation process.

## Figures and Tables

**Table 1:** The Zülhke macroscopic classification.

Groups	Adhesion scores			
	1	2	3	4
Control (*n* = 12)	2	0	0	0
Abrasion (*n* = 12)	1	3	5	3
Abrasion + colchicine (*n* = 12)	4	5	2	1

**Table 2:** The Zülhke microscopic classification.

Groups	Adhesion scores			
	1	2	3	4
Control (*n* = 12)	2	0	0	0
Abrasion (*n* = 12)	0	3	5	4
Abrasion + colchicine (*n* = 12)	3	4	3	2

## The Source of Study

The source of the study is Department of General Surgery, School of Medicine, Suleyman Demirel University, Isparta, Turkey.
