# Corrigendum to “To Investigate the Effect of Colchicine in Prevention of Adhesions Caused by Serosal Damage in Rats”

**DOI:** 10.1155/2018/5128184

**Published:** 2018-11-01

**Authors:** Ihsan Yıldız, Yavuz Savas Koca, Aziz Kemal Emek, Tekinalp Gelen

**Affiliations:** ^1^Department of General Surgery, School of Medicine, Suleyman Demirel University, Isparta, Turkey; ^2^Department of General Surgery, School of Medicine, Akdeniz University, Antalya, Turkey; ^3^Department of Pathology, School of Medicine, Akdeniz University, Antalya, Turkey

In the article titled “To Investigate the Effect of Colchicine in Prevention of Adhesions Caused by Serosal Damage in Rats” [1], Drs. Aziz Kemal Emek and Tekinalp Gelen were missing from the authors' list. Dr. Aziz Kemal Emek's contribution is the study design, while Dr. Tekinalp Gelen's contribution is the examination of the pathology specimens of the research. The corrected authors' list and affiliations are shown above.
